# Crystal structure of a heterometallic coordination polymer: *catena*-poly[[[tetra­aqua­cobalt(II)]-μ-pyridine-2,6-di­carboxyl­ato-calcium(II)-μ-pyridine-2,6-di­carboxyl­ato] dihydrate]

**DOI:** 10.1107/S2056989018007120

**Published:** 2018-05-18

**Authors:** Jie-Shuang Lin, Bing-Guang Zhang

**Affiliations:** a Key Laboratory of Catalysis and Materials Sciences of the State Ethnic Affairs Commission & Ministry of Education, College of Chemistry and Material Science, South-Central University for Nationalities, Wuhan 430074, People’s Republic of China

**Keywords:** crystal structure, heterometallic complex, cobalt carboxyl­ates, calcium carboxyl­ates, pyridine-2,6-di­carboxyl­ate anions, hydrogen bonds, offset π–π inter­actions

## Abstract

In the crystal of the title polymeric complex, {[CoCa(C_7_H_3_NO_4_)_2_(H_2_O)_4_]·2H_2_O}_*n*_ (**1**), the Co^II^ ion is *N*,*O*,*O*′-chelated by two pyridine-2,6-di­carboxyl­ate anions in a distorted N_2_O_4_ octa­hedral geometry, and two carboxyl­ate O atoms of pyridine-2,6-di­carboxyl­ate anions bridge tetra­aqua­calcium(II) units to form polymeric chains propagating along the *b*-axis direction. In the crystal, O—H⋯O and C—H⋯O hydrogen bonds, and offset π–π stacking inter­actions [inter­centroid distances = 3.551 (1) and 3.746 (1) Å] involving inversion-related pyridine rings link the polymeric chains and lattice water mol­ecules to form a supra­molecular three-dimensional framework.

## Abstract 2

The pyridine-2,6-di­carboxyl­ate anions bridge the Ca^II^ and Co^II^ cations to form a polymeric complex chain propagating along the *b*-axis direction.

## Chemical context   

The controllable synthesis of heterometallic polymers, with their fascinating structures and outstanding properties, is still a challenge in crystal engineering (Cai *et al.*, 2012 ▸; Ma *et al.*, 2014 ▸; Sun *et al.*, 2014 ▸; Ward, 2007 ▸). The influencing factors include the coordination geometry of the metal centre, reaction of solvent, temperature, metal-to-ligand ratio, pH value, the nature of ligand, and so on (Chen *et al.*, 2012 ▸; Guo & Cao, 2009 ▸; Ni *et al.*, 2009 ▸; Yamada *et al.*, 2011 ▸). According to our earlier study (Sun *et al.*, 2016 ▸), heterometallic complexes containing both alkaline earth metals and *d*-block transition metals are available because the former are structurally malleable and they have a strong affinity to O atoms rather than N atoms (Cao *et al.*, 2015 ▸; Yu *et al.*, 2013 ▸), and the latter have a strong tendency to coordinate to both N- and O-atom donors (Hu *et al.*, 2013 ▸; Zhang *et al.*, 2013 ▸). Meanwhile, pyridinedi­carb­oxy­lic acid (H_2_pdc) is widely used in the construction of various metal–organic frameworks for two main reasons. Firstly, the O and N atoms in these ligands made them easy to chelate or bridge metal ions. Secondly, they can be completely or partially deprotonated to generate Hpdc^−^ or pyc^2−^, displaying a variety of coordination modes. As a part of our ongoing studies on heterometallic frameworks, we describe here the synthesis and crystal structure of the title complex,**1**

## Structural commentary   

**Figure 1:**
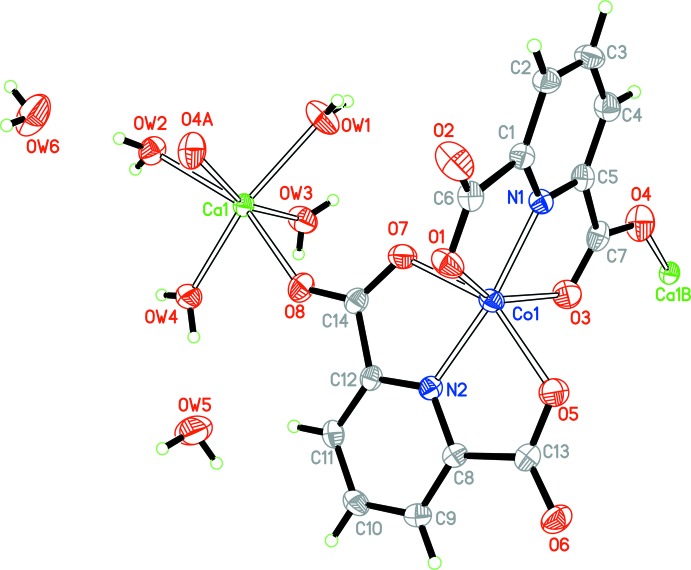
The coordination mode and atom-numbering scheme for the asymmetric unit of **1**. Displacement ellipsoids are drawn at the 50% probability level [symmetry codes: (A) *x*, *y* − 1, *z*; (B) *x*, *y* + 1, *z*].

**Figure 2:**
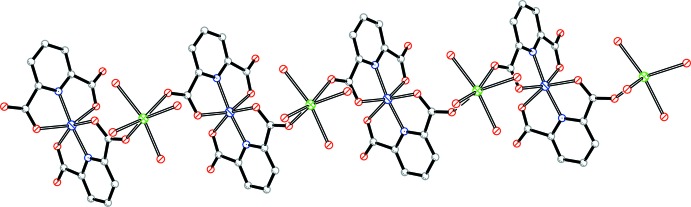
The chain formed by pdc^2−^ anions, and Co^II^ and Ca^II^ centres, propagating along the *b-*axis direction.

The asymmetric unit of **1** contains one cobalt centre, one calcium centre, two pdc^2−^ anions, four coordinated water mol­ecules and two lattice water mol­ecules (Fig. 1 ▸). The Co—O(N) bond lengths are in the range 2.0172 (13)–2.2018 (12) Å and the Ca—O bond lengths are in the range 2.3358 (12)–2.3727 (12) Å (Table 1 ▸). All the data are comparable to those reported for other related Co^II^–pdc and Ca^II^–pdc complexes (Jung *et al.*, 2008 ▸; Shi *et al.*, 2012 ▸). Each Co^II^ centre is chelated by four O and two N atoms from two pdc^2−^ anions, forming a distorted octa­hedral geometry. The mean deviation of the equatorial plane constructed by atoms N1, N2, O5 and O7 is 0.02 Å. Each Ca^II^ centre is six-coordinated by two carboxyl­ate O atoms from two pdc^2−^ anions and four water mol­ecules, displaying a distorted octa­hedron (Fig. 1 ▸). The mean deviation of the equatorial plane constructed by atoms O4, O*W*1, O*W*3 and O*W*4 is 0.08 Å. The CoN_2_O_4_ and CaO_6_ polyhedra are linked by pdc^2−^ anions to form polymeric chains along the *b-*axis direction (Fig. 2 ▸).
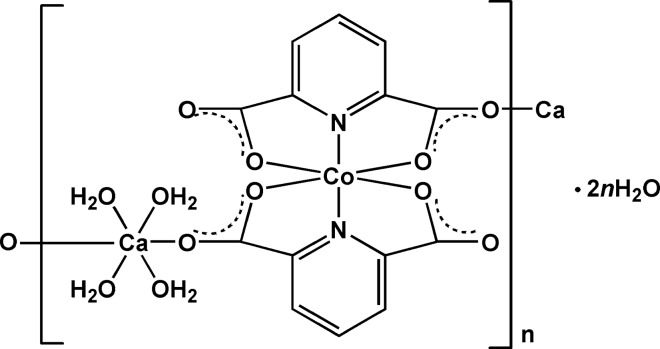


## Supra­molcular features   

**Figure 3:**
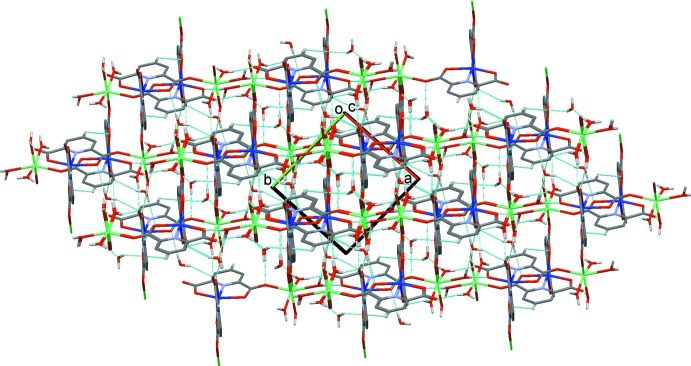
A view along the *c* axis of the crystal packing of **1**. The hydrogen bonds are shown as dashed lines (see Table 2 ▸). For clarity, only the H atoms involved in these inter­actions have been included.

In the crystal of **1**, the polymeric chains are linked by O—H⋯O and C—H⋯O hydrogen bonds involving the water mol­ecules and carboxyl groups, so forming a supra­molecular three-dimensional framework (Table 2 ▸ and Fig. 3 ▸). Within the framework, inversion-related pyridine rings are linked by offset π–π inter­actions reinforcing the framework: *Cg*5⋯*Cg*5^vii^ = 3.746 (1) Å, inter­planar distance = 3.309 (1) Å, slippage = 1.755 Å; *Cg*6⋯*Cg*6^viii^ = 3.551 (1) Å, inter­planar distance = 3.279 (1) Å, slippage = 1.363 Å; *Cg*5 and *Cg*6 are the centroids of pyridine rings N1/C1–C5 and N2/C8–C12, respectively; symmetry codes: (vii) −*x* + 1, −*y* + 1, −*z*; (viii) −*x* + 1, −*y*, −*z* + 1.

## Database survey   

A search of the Cambridge Structural Database (Version 5.39, last update February 2018; Groom *et al.*, 2016 ▸) for cobalt complexes of the ligand pyridine-2,6-di­carb­oxy­lic acid gave 180 hits, of which 58 are polymeric complexes. They include a number of alkali metal heterometallic coordination polymes, four involving K^+^ and seven Na^+^, but no alkali earth metal heterometallic coordination polymers. Hence, the title compound **1** is the first reported heterometallic coordination polymer involving the ligand pyridine-2,6-di­carb­oxy­lic acid, Co^II^ and an alkali earth metal (Ca^II^).

## Synthesis and crystallization   

A mixture of H_2_pdc (167 mg, 1 mmol), Co(CH_3_COO)_2_·4H_2_O (125 mg, 0.5 mmol) and CaCl_2_ (110 mg, 1 mmol) in 15 ml of distilled H_2_O was stirred for 10 min in air. 0.5 *M* NaOH was added dropwise and the mixture was turned into a Parr Teflon-lined stainless steel vessel and heated at 423 K for 3 d. Blue *[purple in CIF?]* block-shaped crystals of **1** were obtained in a yield of 70% (based on pyridine-2,6-di­carb­oxy­lic acid).

## Refinement   

Crystal data, data collection and structure refinement details are summarized in Table 3 ▸. The H atoms of the water mol­ecules were located from difference-Fourier maps and refined with distance restraints: O—H = 0.85 (1) Å, H⋯H = 1.34 (1) Å with *U*_iso_(H) = 1.5*U*_eq_(O). C-bound H atoms atoms were included in calculated positions and refined as riding: C—H = 0.93 Å with *U*_iso_(H) = 1.2*U*_eq_(C).

## Supplementary Material

Crystal structure: contains datablock(s) 1, global. DOI: 10.1107/S2056989018007120/xu5921sup1.cif

CCDC reference: 1832782

Additional supporting information:  crystallographic information; 3D view; checkCIF report

## Figures and Tables

**Table 1:** Selected bond lengths (Å)

Co1—N1	2.0172 (13)	Ca1—O4^i^	2.3358 (12)
Co1—N2	2.0199 (13)	Ca1—O*W*4	2.3449 (13)
Co1—O5	2.1466 (12)	Ca1—O8	2.3458 (12)
Co1—O3	2.1469 (13)	Ca1—O*W*1	2.3476 (13)
Co1—O1	2.1643 (12)	Ca1—O*W*3	2.3719 (13)
Co1—O7	2.2018 (12)	Ca1—O*W*2	2.3727 (12)

Symmetry code: (i) 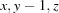.

**Table 2:** Hydrogen-bond geometry (Å, °)

*D*—H⋯*A*	*D*—H	H⋯*A*	*D*⋯*A*	*D*—H⋯*A*
O*W*1—H*W*1*A*⋯O2^ii^	0.84 (1)	1.93 (1)	2.769 (2)	171 (3)
O*W*1—H*W*1*B*⋯O2^iii^	0.85 (1)	2.06 (1)	2.870 (2)	161 (3)
O*W*2—H*W*2*A*⋯O*W*6	0.85 (1)	2.00 (1)	2.846 (2)	175 (3)
O*W*2—H*W*2*B*⋯O5^iv^	0.85 (1)	1.89 (1)	2.730 (2)	173 (3)
O*W*3—H*W*3*A*⋯O1^ii^	0.84 (1)	1.99 (1)	2.817 (2)	172 (3)
O*W*3—H*W*3*B*⋯O6^v^	0.84 (1)	2.12 (1)	2.923 (2)	162 (3)
O*W*4—H*W*4*A*⋯O6^iv^	0.84 (1)	2.02 (1)	2.851 (2)	172 (3)
O*W*4—H*W*4*B*⋯O*W*5	0.84 (1)	1.90 (1)	2.741 (2)	173 (3)
O*W*5—H*W*5*A*⋯O8^vi^	0.85 (1)	2.10 (1)	2.946 (2)	174 (3)
O*W*5—H*W*5*B*⋯O3^v^	0.85 (1)	2.08 (2)	2.870 (2)	153 (3)
O*W*6—H*W*6*A*⋯O7^i^	0.84 (1)	2.13 (1)	2.945 (2)	163 (3)
O*W*6—H*W*6*B*⋯O2^iv^	0.84 (1)	2.34 (1)	3.140 (2)	160 (3)
C2—H2*A*⋯O7^iii^	0.93	2.56	3.448 (2)	160
C10—H10*A*⋯O3^v^	0.93	2.55	3.246 (2)	132

Symmetry codes: (i) 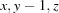; (ii) 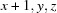; (iii) 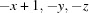; (iv) 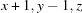; (v) 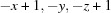; (vi) 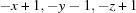.

**Table 3:** Experimental details

Crystal data	
Chemical formula	[CaCo(C_7_H_3_NO_4_)_2_(H_2_O)_4_]·2H_2_O
*M*_r_	537.31
Crystal system, space group	Triclinic, *P* 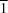
Temperature (K)	296
*a*, *b*, *c* (Å)	8.6299 (8), 8.7781 (8), 14.0726 (12)
α, β, γ (°)	80.683 (1), 73.602 (1), 89.568 (1)
*V* (Å^3^)	1008.38 (16)
*Z*	2
Radiation type	Mo *K*α
μ (mm^−1^)	1.18
Crystal size (mm)	0.35 × 0.33 × 0.33

Data collection	
Diffractometer	Bruker SMART CCD
No. of measured, independent and observed [*I* > 2σ(*I*)] reflections	7052, 3537, 3342
*R*_int_	0.012
(sin θ/λ)_max_ (Å^−1^)	0.595

Refinement	
*R*[*F* ^2^ > 2σ(*F* ^2^)], *wR*(*F* ^2^), *S*	0.023, 0.064, 1.01
No. of reflections	3537
No. of parameters	326
No. of restraints	18
H-atom treatment	H atoms treated by a mixture of independent and constrained refinement
Δρ_max_, Δρ_min_ (e Å^−3^)	0.44, −0.49

Computer programs: *APEX2* and *SAINT* (Bruker, 2009 ▸), *SHELXS97* and *SHELXTL* (Sheldrick, 2008 ▸), *SHELXL2014* (Sheldrick, 2015 ▸), *Mercury* (Macrae *et al.*, 2008 ▸) and *publCIF* (Westrip, 2010 ▸).

## supplementary crystallographic information

### Crystal data

**Table:**

[CaCo(C_7_H_3_NO_4_)_2_(H_2_O)_4_]·2H_2_O	*Z* = 2
*M**_r_* = 537.31	*F*(000) = 550
Triclinic, *P*1	*D*_x_ = 1.770 Mg m^−^^3^
Hall symbol: -P 1	Mo *K*α radiation, λ = 0.71073 Å
*a* = 8.6299 (8) Å	Cell parameters from 5842 reflections
*b* = 8.7781 (8) Å	θ = 2.4–27.7°
*c* = 14.0726 (12) Å	µ = 1.18 mm^−^^1^
α = 80.683 (1)°	*T* = 296 K
β = 73.602 (1)°	Block, purple
γ = 89.568 (1)°	0.35 × 0.33 × 0.33 mm
*V* = 1008.38 (16) Å^3^	

### Data collection

**Table:**

Bruker SMART CCD diffractometer	3342 reflections with *I* > 2σ(*I*)
Radiation source: fine-focus sealed tube	*R*_int_ = 0.012
Graphite monochromator	θ_max_ = 25.0°, θ_min_ = 2.6°
φ and ω scans	*h* = −10→10
7052 measured reflections	*k* = −10→9
3537 independent reflections	*l* = −16→16

### Refinement

**Table:**

Refinement on *F*^2^	Secondary atom site location: difference Fourier map
Least-squares matrix: full	Hydrogen site location: mixed
*R*[*F*^2^ > 2σ(*F*^2^)] = 0.023	H atoms treated by a mixture of independent and constrained refinement
*wR*(*F*^2^) = 0.064	*w* = 1/[σ^2^(*F*_o_^2^) + (0.0385*P*)^2^ + 0.4728*P*] where *P* = (*F*_o_^2^ + 2*F*_c_^2^)/3
*S* = 1.01	(Δ/σ)_max_ = 0.001
3537 reflections	Δρ_max_ = 0.44 e Å^−^^3^
326 parameters	Δρ_min_ = −0.49 e Å^−^^3^
18 restraints	Extinction correction: SHELXL2014 (Sheldrick, 2015), Fc^*^=kFc[1+0.001xFc^2^λ^3^/sin(2θ)]^-1/4^
Primary atom site location: structure-invariant direct methods	Extinction coefficient: 0.0300 (14)

### Special details

**Table:**

Geometry. All esds (except the esd in the dihedral angle between two l.s. planes) are estimated using the full covariance matrix. The cell esds are taken into account individually in the estimation of esds in distances, angles and torsion angles; correlations between esds in cell parameters are only used when they are defined by crystal symmetry. An approximate (isotropic) treatment of cell esds is used for estimating esds involving l.s. planes.
Refinement. Refinement of F^2^ against ALL reflections. The weighted R-factor wR and goodness of fit S are based on F^2^, conventional R-factors R are based on F, with F set to zero for negative F^2^. The threshold expression of F^2^ > 2sigma(F^2^) is used only for calculating R-factors(gt) etc. and is not relevant to the choice of reflections for refinement. R-factors based on F^2^ are statistically about twice as large as those based on F, and R- factors based on ALL data will be even larger.

### Fractional atomic coordinates and isotropic or equivalent isotropic displacement parameters (Å^2^)

**Table:**

	*x*	*y*	*z*	*U*_iso_*/*U*_eq_	
Co1	0.39269 (3)	0.14153 (3)	0.252249 (15)	0.02155 (10)	
Ca1	0.86691 (4)	−0.38042 (3)	0.24956 (2)	0.01922 (10)	
O1	0.24638 (15)	0.02836 (14)	0.18004 (9)	0.0291 (3)	
O2	0.20443 (16)	0.02565 (16)	0.03080 (10)	0.0364 (3)	
O3	0.55173 (16)	0.32701 (14)	0.25445 (9)	0.0301 (3)	
O4	0.73160 (16)	0.50764 (15)	0.15426 (11)	0.0351 (3)	
O5	0.19108 (15)	0.25552 (15)	0.33569 (9)	0.0308 (3)	
O6	0.02552 (14)	0.25623 (14)	0.48985 (9)	0.0297 (3)	
O7	0.57745 (15)	−0.03428 (14)	0.24055 (9)	0.0295 (3)	
O8	0.63798 (15)	−0.25231 (14)	0.32698 (10)	0.0325 (3)	
N1	0.47499 (16)	0.24083 (15)	0.10671 (10)	0.0192 (3)	
N2	0.33969 (16)	0.01759 (15)	0.39227 (10)	0.0192 (3)	
C1	0.42012 (19)	0.18591 (18)	0.03843 (12)	0.0200 (3)	
C2	0.4907 (2)	0.23423 (19)	−0.06355 (12)	0.0247 (4)	
H2A	0.4515	0.1975	−0.1111	0.030*	
C3	0.6221 (2)	0.3394 (2)	−0.09255 (13)	0.0280 (4)	
H3A	0.6739	0.3713	−0.1604	0.034*	
C4	0.6763 (2)	0.3971 (2)	−0.02081 (13)	0.0263 (4)	
H4A	0.7631	0.4685	−0.0397	0.032*	
C5	0.59765 (19)	0.34531 (18)	0.07952 (12)	0.0209 (3)	
C6	0.27915 (19)	0.06991 (19)	0.08568 (12)	0.0226 (3)	
C7	0.6325 (2)	0.39819 (19)	0.16942 (13)	0.0237 (4)	
C8	0.22421 (19)	0.06489 (18)	0.46591 (12)	0.0194 (3)	
C9	0.1926 (2)	−0.0107 (2)	0.56454 (12)	0.0236 (4)	
H9A	0.1131	0.0227	0.6159	0.028*	
C10	0.2824 (2)	−0.13715 (19)	0.58450 (12)	0.0251 (4)	
H10A	0.2628	−0.1901	0.6498	0.030*	
C11	0.4018 (2)	−0.18510 (19)	0.50709 (12)	0.0232 (3)	
H11A	0.4627	−0.2698	0.5197	0.028*	
C12	0.42759 (19)	−0.10363 (18)	0.41099 (12)	0.0200 (3)	
C13	0.13795 (19)	0.20400 (19)	0.42944 (12)	0.0216 (3)	
C14	0.55835 (19)	−0.13350 (19)	0.31863 (12)	0.0222 (3)	
OW1	0.95691 (18)	−0.19898 (17)	0.10151 (11)	0.0404 (3)	
OW2	1.02789 (16)	−0.59450 (15)	0.21033 (10)	0.0319 (3)	
OW3	1.06339 (17)	−0.23885 (16)	0.29216 (10)	0.0354 (3)	
OW4	0.84002 (17)	−0.51094 (15)	0.41287 (9)	0.0325 (3)	
OW5	0.6149 (3)	−0.4991 (2)	0.59299 (15)	0.0782 (7)	
OW6	0.8857 (2)	−0.8525 (2)	0.16230 (15)	0.0604 (5)	
HW1A	1.039 (2)	−0.139 (3)	0.080 (2)	0.091*	
HW4A	0.902 (3)	−0.577 (3)	0.430 (2)	0.091*	
HW3A	1.126 (3)	−0.163 (2)	0.2603 (17)	0.091*	
HW4B	0.771 (3)	−0.499 (3)	0.4666 (13)	0.091*	
HW3B	1.060 (4)	−0.247 (3)	0.3528 (8)	0.091*	
HW1B	0.898 (3)	−0.168 (3)	0.0638 (19)	0.091*	
HW2A	0.985 (4)	−0.668 (2)	0.192 (2)	0.091*	
HW2B	1.072 (3)	−0.639 (3)	0.2531 (18)	0.091*	
HW6A	0.7978 (18)	−0.902 (3)	0.172 (2)	0.091*	
HW5B	0.596 (4)	−0.447 (3)	0.6408 (17)	0.091*	
HW6B	0.958 (2)	−0.907 (3)	0.134 (2)	0.091*	
HW5A	0.539 (3)	−0.569 (3)	0.613 (2)	0.091*	

### Atomic displacement parameters (Å^2^)

**Table:**

	*U*^11^	*U*^22^	*U*^33^	*U*^12^	*U*^13^	*U*^23^
Co1	0.02474 (14)	0.02310 (14)	0.01634 (14)	0.00250 (9)	−0.00629 (9)	−0.00120 (9)
Ca1	0.01825 (18)	0.01797 (18)	0.02199 (19)	0.00136 (13)	−0.00637 (13)	−0.00374 (13)
O1	0.0303 (7)	0.0314 (7)	0.0239 (6)	−0.0101 (5)	−0.0077 (5)	0.0011 (5)
O2	0.0394 (7)	0.0394 (8)	0.0349 (7)	−0.0162 (6)	−0.0220 (6)	0.0022 (6)
O3	0.0390 (7)	0.0304 (7)	0.0253 (7)	−0.0001 (6)	−0.0148 (6)	−0.0069 (5)
O4	0.0356 (7)	0.0294 (7)	0.0482 (8)	−0.0055 (6)	−0.0228 (6)	−0.0095 (6)
O5	0.0333 (7)	0.0340 (7)	0.0240 (6)	0.0140 (6)	−0.0087 (5)	−0.0014 (5)
O6	0.0273 (6)	0.0335 (7)	0.0293 (7)	0.0119 (5)	−0.0074 (5)	−0.0094 (5)
O7	0.0306 (7)	0.0314 (7)	0.0226 (6)	0.0071 (5)	−0.0027 (5)	−0.0022 (5)
O8	0.0291 (7)	0.0267 (7)	0.0385 (7)	0.0121 (5)	−0.0044 (6)	−0.0060 (6)
N1	0.0195 (7)	0.0200 (7)	0.0191 (7)	0.0001 (5)	−0.0077 (5)	−0.0025 (5)
N2	0.0193 (7)	0.0200 (7)	0.0193 (7)	0.0026 (5)	−0.0068 (5)	−0.0038 (5)
C1	0.0203 (8)	0.0204 (8)	0.0210 (8)	0.0017 (6)	−0.0089 (6)	−0.0035 (6)
C2	0.0296 (9)	0.0265 (9)	0.0205 (8)	0.0037 (7)	−0.0098 (7)	−0.0059 (7)
C3	0.0274 (9)	0.0317 (10)	0.0199 (8)	0.0013 (7)	−0.0005 (7)	−0.0008 (7)
C4	0.0204 (8)	0.0240 (9)	0.0314 (9)	−0.0025 (7)	−0.0042 (7)	−0.0014 (7)
C5	0.0181 (8)	0.0191 (8)	0.0265 (8)	0.0016 (6)	−0.0086 (6)	−0.0034 (6)
C6	0.0212 (8)	0.0206 (8)	0.0276 (9)	0.0003 (7)	−0.0096 (7)	−0.0033 (7)
C7	0.0227 (8)	0.0212 (8)	0.0328 (10)	0.0056 (7)	−0.0152 (7)	−0.0079 (7)
C8	0.0178 (7)	0.0214 (8)	0.0206 (8)	0.0005 (6)	−0.0067 (6)	−0.0059 (6)
C9	0.0230 (8)	0.0276 (9)	0.0198 (8)	0.0004 (7)	−0.0046 (7)	−0.0051 (7)
C10	0.0296 (9)	0.0252 (9)	0.0199 (8)	−0.0034 (7)	−0.0086 (7)	0.0010 (7)
C11	0.0256 (8)	0.0186 (8)	0.0268 (9)	0.0016 (7)	−0.0109 (7)	−0.0012 (7)
C12	0.0195 (8)	0.0180 (8)	0.0239 (8)	0.0007 (6)	−0.0076 (6)	−0.0050 (6)
C13	0.0206 (8)	0.0230 (8)	0.0244 (8)	0.0025 (7)	−0.0096 (7)	−0.0074 (7)
C14	0.0203 (8)	0.0215 (8)	0.0261 (9)	0.0011 (7)	−0.0073 (7)	−0.0069 (7)
OW1	0.0403 (8)	0.0436 (8)	0.0358 (8)	−0.0165 (7)	−0.0177 (6)	0.0116 (6)
OW2	0.0345 (7)	0.0307 (7)	0.0338 (7)	0.0123 (6)	−0.0144 (6)	−0.0069 (6)
OW3	0.0389 (8)	0.0386 (8)	0.0292 (7)	−0.0145 (6)	−0.0115 (6)	−0.0036 (6)
OW4	0.0392 (8)	0.0295 (7)	0.0255 (7)	0.0071 (6)	−0.0057 (6)	−0.0014 (5)
OW5	0.0881 (15)	0.0622 (12)	0.0629 (12)	−0.0295 (10)	0.0275 (10)	−0.0355 (10)
OW6	0.0414 (9)	0.0520 (10)	0.0866 (13)	−0.0007 (8)	−0.0021 (9)	−0.0370 (9)

### Geometric parameters (Å, º)

**Table:**

Co1—N1	2.0172 (13)	C2—H2A	0.9300
Co1—N2	2.0199 (13)	C3—C4	1.389 (3)
Co1—O5	2.1466 (12)	C3—H3A	0.9300
Co1—O3	2.1469 (13)	C4—C5	1.384 (2)
Co1—O1	2.1643 (12)	C4—H4A	0.9300
Co1—O7	2.2018 (12)	C5—C7	1.521 (2)
Ca1—O4^i^	2.3358 (12)	C8—C9	1.390 (2)
Ca1—OW4	2.3449 (13)	C8—C13	1.519 (2)
Ca1—O8	2.3458 (12)	C9—C10	1.386 (2)
Ca1—OW1	2.3476 (13)	C9—H9A	0.9300
Ca1—OW3	2.3719 (13)	C10—C11	1.390 (2)
Ca1—OW2	2.3727 (12)	C10—H10A	0.9300
O1—C6	1.268 (2)	C11—C12	1.382 (2)
O2—C6	1.243 (2)	C11—H11A	0.9300
O3—C7	1.266 (2)	C12—C14	1.520 (2)
O4—C7	1.242 (2)	OW1—HW1A	0.844 (10)
O4—Ca1^ii^	2.3358 (12)	OW1—HW1B	0.846 (10)
O5—C13	1.274 (2)	OW2—HW2A	0.849 (10)
O6—C13	1.236 (2)	OW2—HW2B	0.845 (10)
O7—C14	1.258 (2)	OW3—HW3A	0.838 (10)
O8—C14	1.253 (2)	OW3—HW3B	0.837 (10)
N1—C5	1.334 (2)	OW4—HW4A	0.840 (10)
N1—C1	1.338 (2)	OW4—HW4B	0.842 (10)
N2—C8	1.338 (2)	OW5—HW5B	0.854 (10)
N2—C12	1.337 (2)	OW5—HW5A	0.854 (10)
C1—C2	1.387 (2)	OW6—HW6A	0.843 (10)
C1—C6	1.517 (2)	OW6—HW6B	0.839 (10)
C2—C3	1.392 (3)		
			
N1—Co1—N2	170.56 (5)	C4—C3—H3A	119.8
N1—Co1—O5	113.11 (5)	C2—C3—H3A	119.8
N2—Co1—O5	76.33 (5)	C5—C4—C3	118.16 (16)
N1—Co1—O3	76.31 (5)	C5—C4—H4A	120.9
N2—Co1—O3	104.44 (5)	C3—C4—H4A	120.9
O5—Co1—O3	89.74 (5)	N1—C5—C4	120.99 (15)
N1—Co1—O1	76.52 (5)	N1—C5—C7	112.31 (14)
N2—Co1—O1	103.75 (5)	C4—C5—C7	126.69 (15)
O5—Co1—O1	93.20 (5)	O2—C6—O1	125.55 (15)
O3—Co1—O1	151.54 (5)	O2—C6—C1	118.66 (15)
N1—Co1—O7	94.39 (5)	O1—C6—C1	115.77 (14)
N2—Co1—O7	76.18 (5)	O4—C7—O3	125.91 (16)
O5—Co1—O7	152.44 (5)	O4—C7—C5	118.68 (16)
O3—Co1—O7	95.18 (5)	O3—C7—C5	115.38 (14)
O1—Co1—O7	95.16 (5)	N2—C8—C9	120.75 (15)
O4^i^—Ca1—OW4	116.69 (5)	N2—C8—C13	113.26 (13)
O4^i^—Ca1—O8	92.96 (5)	C9—C8—C13	126.00 (14)
OW4—Ca1—O8	84.24 (5)	C10—C9—C8	118.39 (15)
O4^i^—Ca1—OW1	82.87 (5)	C10—C9—H9A	120.8
OW4—Ca1—OW1	160.32 (5)	C8—C9—H9A	120.8
O8—Ca1—OW1	97.60 (5)	C9—C10—C11	120.17 (15)
O4^i^—Ca1—OW3	160.85 (5)	C9—C10—H10A	119.9
OW4—Ca1—OW3	80.10 (5)	C11—C10—H10A	119.9
O8—Ca1—OW3	98.14 (5)	C12—C11—C10	118.30 (15)
OW1—Ca1—OW3	80.24 (5)	C12—C11—H11A	120.8
O4^i^—Ca1—OW2	78.31 (5)	C10—C11—H11A	120.8
OW4—Ca1—OW2	80.75 (5)	N2—C12—C11	121.15 (15)
O8—Ca1—OW2	156.81 (5)	N2—C12—C14	113.20 (14)
OW1—Ca1—OW2	102.49 (5)	C11—C12—C14	125.58 (14)
OW3—Ca1—OW2	96.57 (5)	O6—C13—O5	125.96 (15)
C6—O1—Co1	115.23 (10)	O6—C13—C8	119.55 (15)
C7—O3—Co1	115.79 (10)	O5—C13—C8	114.48 (14)
C7—O4—Ca1^ii^	136.36 (12)	O8—C14—O7	126.01 (15)
C13—O5—Co1	116.59 (10)	O8—C14—C12	117.73 (15)
C14—O7—Co1	114.36 (10)	O7—C14—C12	116.25 (14)
C14—O8—Ca1	144.69 (12)	Ca1—OW1—HW1A	131.0 (19)
C5—N1—C1	121.46 (14)	Ca1—OW1—HW1B	123.2 (19)
C5—N1—Co1	118.88 (11)	HW1A—OW1—HW1B	104.7 (15)
C1—N1—Co1	119.07 (11)	Ca1—OW2—HW2A	117 (2)
C8—N2—C12	121.24 (14)	Ca1—OW2—HW2B	118 (2)
C8—N2—Co1	119.12 (11)	HW2A—OW2—HW2B	104.6 (15)
C12—N2—Co1	119.49 (11)	Ca1—OW3—HW3A	132.0 (19)
N1—C1—C2	120.97 (15)	Ca1—OW3—HW3B	118.4 (19)
N1—C1—C6	112.71 (14)	HW3A—OW3—HW3B	108.0 (15)
C2—C1—C6	126.33 (15)	Ca1—OW4—HW4A	126.5 (19)
C1—C2—C3	117.92 (15)	Ca1—OW4—HW4B	128.2 (18)
C1—C2—H2A	121.0	HW4A—OW4—HW4B	105.2 (15)
C3—C2—H2A	121.0	HW5B—OW5—HW5A	103.9 (15)
C4—C3—C2	120.46 (16)	HW6A—OW6—HW6B	105.7 (15)

Symmetry codes: (i) *x*, *y*−1, *z*; (ii) *x*, *y*+1, *z*.

### Hydrogen-bond geometry (Å, º)

**Table:**

*D*—H···*A*	*D*—H	H···*A*	*D*···*A*	*D*—H···*A*
O*W*1—H*W*1*A*···O2^iii^	0.84 (1)	1.93 (1)	2.769 (2)	171 (3)
O*W*1—H*W*1*B*···O2^iv^	0.85 (1)	2.06 (1)	2.870 (2)	161 (3)
O*W*2—H*W*2*A*···O*W*6	0.85 (1)	2.00 (1)	2.846 (2)	175 (3)
O*W*2—H*W*2*B*···O5^v^	0.85 (1)	1.89 (1)	2.730 (2)	173 (3)
O*W*3—H*W*3*A*···O1^iii^	0.84 (1)	1.99 (1)	2.817 (2)	172 (3)
O*W*3—H*W*3*B*···O6^vi^	0.84 (1)	2.12 (1)	2.923 (2)	162 (3)
O*W*4—H*W*4*A*···O6^v^	0.84 (1)	2.02 (1)	2.851 (2)	172 (3)
O*W*4—H*W*4*B*···O*W*5	0.84 (1)	1.90 (1)	2.741 (2)	173 (3)
O*W*5—H*W*5*A*···O8^vii^	0.85 (1)	2.10 (1)	2.946 (2)	174 (3)
O*W*5—H*W*5*B*···O3^vi^	0.85 (1)	2.08 (2)	2.870 (2)	153 (3)
O*W*6—H*W*6*A*···O7^i^	0.84 (1)	2.13 (1)	2.945 (2)	163 (3)
O*W*6—H*W*6*B*···O2^v^	0.84 (1)	2.34 (1)	3.140 (2)	160 (3)
C2—H2*A*···O7^iv^	0.93	2.56	3.448 (2)	160
C10—H10*A*···O3^vi^	0.93	2.55	3.246 (2)	132

Symmetry codes: (i) *x*, *y*−1, *z*; (iii) *x*+1, *y*, *z*; (iv) −*x*+1, −*y*, −*z*; (v) *x*+1, *y*−1, *z*; (vi) −*x*+1, −*y*, −*z*+1; (vii) −*x*+1, −*y*−1, −*z*+1.
